# Early psychosocial interventions after disasters, terrorism and other shocking events: is there a gap between norms and practice in Europe?

**DOI:** 10.3402/ejpt.v4i0.19093

**Published:** 2013-02-05

**Authors:** Hans Te Brake, Michel Dückers

**Affiliations:** Impact, National knowledge and advice centre for psychosocial care concerning critical incidents, Partner in Arq, Psychotrauma Expert Group, Diemen, The Netherlands

**Keywords:** Early psychosocial interventions, disaster, shocking events, psychological assessment, cross-cultural comparison

## Abstract

**Background:**

Internationally, several initiatives exist to describe standards for post-disaster psychosocial care.

**Objective:**

This study explored the level of consensus of experts within Europe on a set of recommendations on early psychosocial intervention after shocking events (Dutch guidelines), and to what degree these standards are implemented into mental health care practice.

**Methods:**

Two hundred and six (mental) health care professionals filled out a questionnaire to assess the extent to which they consider the guidelines’ scope and recommendations relevant and part of the regular practice in their own country. Forty-five European experts from 24 EU countries discussed the guidelines at an international seminar.

**Results:**

The data suggest overall agreement on the standards although many of the recommendations appear not (yet) to be embedded in everyday practice.

**Conclusions:**

Although large consensus exists on standards for early psychosocial care, a chasm between norms and practice appears to exist throughout the EU, stressing the general need for investments in guideline development and implementation.

Although research shows that most victims of disasters, terrorism or other shocking events recover on their own merit, a sizeable amount of them develop long-term disaster-related problems (Norris, Friedman, & Watson, 2002). This important conclusion emphasises the necessity for timely and appropriate psychosocial help for disaster victims. High quality standards and guidance for care providers ideally uses evidence-based procedures. As early as 2001, Seynaeve and colleagues published a policy paper on psychosocial support in situations of mass emergency (Seynaeve, 2001), indicating the need for such standards. Since then, several initiatives have been taken to arrive at standards for psychosocial care after disasters and shocking events. The National Institute for Clinical Excellence (NICE) of the British National Health Services developed guidelines to deal with post-traumatic stress disorder (PTSD) (NICE, 2005). Other examples are the NATO-guidelines on psychosocial care for people affected by disasters and major incidents (NATO Joint Medical Committee, 2008), the “mental health first aid guidelines” on how members of the public can support people affected by a traumatic event (Kelly, Jorm, & Kitchener, 2010), and the PTSD guidelines from the Australian Centre for Post-traumatic Mental Health (ACPMH, 2007; Forbes et al., 2007). In the Netherlands, the “Multidisciplinary guidelines on early psychosocial interventions after disasters, terrorism, and other shocking events” were developed (hereafter dubbed “the Dutch guidelines” – Te Brake et al., 2009).

Given all these initiatives, it would be interesting to see to what degree they reflect commonly held beliefs and grounding principles on the provision of early psychosocial care. Recent European initiatives to establish such standards are The European Network for Traumatic Stress (TENTS) and the project European Guidelines for Target group oriented psychosocial Aftercare (EUTOPA). TENTS aimed to develop European-wide networks of expertise on psychosocial care and post-traumatic stress management. TENTS used a Delphi-method to arrive at consensus on a number of conclusions (Bisson et al., 2010; Witteveen et al., 2012). The current article is a product of the EUTOPA project, funded by the European Committee,^1^ which was also focussed on developing a European perspective on early post-disaster psychosocial care based on agreement among experts. In contrast to the TENTS project, the starting point in EUTOPA was an existing set of guidelines. The aforementioned Dutch guidelines served as a vantage point for two reasons. First, these guidelines were the first guidelines on early psychosocial care within Europe, developed nationally in cooperation with groups of professional end-users; which is important because the chances of successful guideline implementation increase if professionals affected are involved (Eccles, Grimshaw, Shekelle, Schünemann, & Woolf, 2012). Second, the Dutch guidelines are largely build upon insights from the several initiatives mentioned above – there is, for instance, a large overlap with the ACPMH guidelines in its basis on international literature and its recommendations (Taal, 2008). Box 1 contains information on the development of the Dutch guidelines.

***Box 1*. Background of the Dutch evidence-based guidelines on early psychosocial interventions after disasters, terrorism and other shocking events**

The Dutch guidelines were developed in accordance with the methods of multidisciplinary guideline development in mental health care and under the auspices of the Dutch National Steering Committee on Multidisciplinary Guideline Development (Te Brake et al., 2009). Guideline development was financed by the Dutch Ministry of Health, Welfare and Sport and the process was coordinated by Impact, the Dutch national knowledge and advice centre for post-disaster psychosocial care. The systematic development of the Dutch guidelines is consistent with ‘clinical practice guidelines’, as described by Forbes et al. (2010). The recommendations are therefore based, where possible, on research evidence, supplemented with expert group consensus and interpretation (in which additional aspects were also taken into account, such as the preferences of victims, costs, availability, and organisational aspects). A multidisciplinary national panel of experts was formed to develop the guidelines, consisting of 21 members from key organisations involved in early psychosocial care. These included: Mental Healthcare Nursing Federation; Netherlands Psychiatric Association; Dutch Association for Psychotherapy; Netherlands Institute of Psychologists; Dutch College of General Practitioners; Dutch Association of Primary Care Psychologists; Military Mental Health Care Institute of the Ministry of Defence; Netherlands Association of Policy, Management and Research Physicians; Netherlands Association of Social Workers; Netherlands Association of Fire and Disaster Control Services; Netherlands Society of Physicians in Occupational Health; Dutch Association of Behavioural and Cognitive Therapy; Institute for Psychotrauma; Immediate Relief and Aftercare; Victim Support; Council of Regional Medical Officers; Regional Department of Emergency and Disaster Medicine Preparedness; Police Academy of the Netherlands. By including these organisations in the development process, early acceptation by practitioners in the field was accounted for. The procedure resulted in 36 recommendations using the results of an extensive systematic literature search. Te Brake et al. (2009) provide a detailed description of the procedure, as well as the recommendations. The Dutch guidelines are largely consistent with insights from the several international initiatives for standardization. In fact, Taal (2008) showed the Dutch guidelines and the ACPMH guidelines are based on the same international scientific publications and encompass similar recommendations.

The primary goal of this study is to provide insight into the degree of consensus on the grounding principles of early psychosocial interventions. The second aim concerns guideline implementation. As described by Engelhart (2012), the academization of mental health care (or valorisation of scientific knowledge) is receiving increased attention. In the context of the EUTOPA project, we assessed the extent to which evidence-based principles described in the Dutch guidelines have taken root in different countries in Europe. The combination of both aims provides an opportunity to explore whether a typical phenomenon in evidence-based service provision – the well-known gap between norms and practice (Grol, 2001) – also exists in the field of post-disaster psychosocial care in Europe. How do European experts and health care professionals from different countries view “ought” and “is” and do they differ from each other? What early psychosocial interventions are considered to be valuable, relevant and normal practice by European experts?

## Methods

### Procedures and study participants

A total of 161 health care professionals in the Netherlands experienced in the field of (acute) psychosocial care received a standardised questionnaire (see below) during seven meetings about the guidelines, which took place between October 2007 and November 2008. These meetings were held for Community Health Services (GGD), Victim Support Netherlands, acute hospital care staff, psychosocial crisis managers, and school psychologists. In addition, a total of 45 European experts from 24 countries were asked to fill out the questionnaire and were present at the expert meeting (see below). These experts represented (mental) health care professionals in general, including both policy makers and (acute) caregivers (including volunteers). It was our goal to include experts from as many EU member states as possible to maximize the diversity in perspectives. Therefore, one or two experts from each country were invited. We approached experts within our own international network and extended the number of invited experts by using a “snowball approach”; experts were asked to inform us about the experts in their network.

### Questionnaire on suitability and implementation of guideline recommendations

A standardized questionnaire was developed to investigate how respondents weigh the suitability of the standards described in the Dutch guidelines for early interventions (thereby addressing the first aim of this study). In addition, the questionnaire investigated on the respondents’ opinion on the extent to which these standards are customary (in order to explore the potential gap between norm and practice; our second aim).

In total, 31 items were used in the questionnaire addressing the recommendations in the Dutch guidelines (Te Brake et al., 2009; Box 1). For each recommendation, the perceived suitability as a standard was assessed on a Likert-scale from 1 to 5 (respectively “completely disagree”, “somewhat disagree”, “no opinion”, “somewhat agree”, and “completely agree”). Scores were later recoded into three categories: “no” (score 1 or 2, i.e., the respondent disagrees with the statement), “not sure” (score 3), or “yes” (score 4 or 5, i.e., the respondent agrees). In addition, for each recommendation the respondent was asked to indicate the extent to which (on the same 5-point Likert-scale) he or she believes this is already part of everyday practice (with numbers respectively indicating “never brought into practice”, “sometimes brought into practice”, “don't know”, “often brought into practice”, and “always brought into practice”). Again, these scores were later recoded into three categories (“no”, “not sure”, and “yes”).

Not all recommendations could readily be rephrased into questionnaire items. Several recommendations specifically advise further research on a specific topic – most of these (which together constitute a “research agenda”) were left out the questionnaire. On the other hand, some recommendations were rephrased into multiple items. For instance, the recommendation for providing a supportive context (recommendation 10, see Te Brake et al., 2009) outlines five aspects, each of which was rephrased into a separate item (see Table 3).

### European experts consultation

In addition to the questionnaire, the recommendations were discussed among European experts. In September 2008, an international conference took place in Amsterdam within the context of the 2-year EUTOPA-project. During the conference, experts were gathered to evaluate the standards provided in the Dutch guidelines on their relevance and applicability in Europe.

## Results

### Response

During the expert meeting, the questionnaire was filled out by 27 European experts from 20 countries (response rate 62.2%). Respondents from The Netherlands consisted of 89 health care professionals, contacted on seven separate occasions (response rates varied between 35, 5 and 100%, overall response rate was 55.3%). Results are summarised in Tables 1–6, following the outline of the Dutch guidelines: (1) aims of early psychosocial interventions, (2) screening, (3) supportive context, (4) preventive interventions, (5) curative interventions, and (6) organisation of early psychosocial care.

**Table 1:** Agreement and practice: the aims of early psychosocial interventions (total N and relative percentages)

	Agreement								Practice							
		
	European Union				Netherlands				European Union				Netherlands			
				
	N	No	?	Yes	N	No	?	Yes	N	No	?	Yes	N	No	?	Yes
Aims of early psychosocial interventions:																
(i) to promote natural recovery and the use of natural resources	27	0	0	100	87	3	0	97	26	15	4	81	73	6	8	86
(ii) to identify victims who need acute psychological help	27	0	4	96	85	1	0	99	25	24	8	68	71	1	16	83
(iii) to refer and, if necessary, to treat victims who need acute psychosocial help	27	0	7	93	85	5	1	94	26	23	15	62	72	10	8	82

### On the aims of early psychosocial interventions

Table 1 summarizes the results concerning adherence to the aims of early psychosocial interventions as stated in the Dutch guidelines. These are agreed on by most European experts and Dutch health care professionals, and are also often brought into practice, albeit to a lesser degree. During the expert discussions it was agreed that early psychosocial intervention guidelines could stimulate good practice and provide support for the different EU member states. However, guidelines should be interpreted not as stringent protocols, but as a tool for providing guidance to member states in shaping early psychosocial interventions. In this sense, the term “guidance” was preferred to “guidelines.” Furthermore, the actual adoption of guidelines is dependent on the developmental stage of psychosocial care in specific countries. While many of the member states have taken initiatives for national early psychosocial care, the capacity and structure necessary to actually implement these initiatives is not always present.

### On screening

Table 2 shows the results concerning the recommendations on screening. Agreement here is also high (and practice substantially lower), except for the latter two (reversely formulated) items, where less consistency was found. The debate about these items, which deals with population-wide screening, was reflected in the discussions among experts. It was acknowledged that further studies are needed into the effectiveness of population-wide screening during the first weeks after a traumatic event. Negative effects and the most appropriate timing must be investigated before a large-scale survey is used for the purpose of population-wide screening. In addition, further research is needed on the usability of screening instruments based on risk factors for the early tracing of those affected with a high risk of developing trauma-related mental disorders. Also, additional points of interest were identified. First, there are challenges in terms of outreach and reaching the people who need help most – the media were considered useful in this respect. Second, there is a need for appropriate tools for different cultures and situations. Finally, the cost-effectiveness of screening also requires attention.

**Table 2:** Agreement and practice: screening (total N and relative percentages)

	Agreement								Practice							
		
	European Union				Netherlands				European Union				Netherlands			
				
	N	No	?	Yes	N	No	?	Yes	N	No	?	Yes	N	No	?	Yes
For victims with an acute stress disorder, a follow-up meeting is planned for further observation	26	4	0	96	77	8	7	86	25	32	20	48	66	9	27	64
When children and adolescents are screened for symptoms of an acute stress disorder following a shocking event, information is gathered both from the child and from parents/caregivers	26	0	0	100	80	1	9	90	25	28	8	64	68	10	24	66
Victims at increased risk of post-traumatic stress disorder (PTSD) are identified using PTSD questionnaires^a^	26	31	12	58	68	34	27	40	25	72	20	8	60	55	20	25
Victims at increased risk of post-traumatic stress disorder (PTSD) are identified by diagnosis of an acute stress disorder^a^	26	19	12	69	71	14	18	68	25	52	24	24	63	14	27	59

a Negatively formulated recommendations: these items are inconsistent with the guidelines.

### On providing a supportive context

From both the questionnaires (results shown in Table 3) as well as the expert discussions, a strong consensus emerged on the recommendation to provide a supportive context. This should enhance the powers of recovery among those affected.

**Table 3:** Agreement and practice: the components of a supportive context (Total N and relative percentages)

	Agreement								Practice							
		
	European Union				Netherlands				European Union				Netherlands			
				
A supportive context is offered, consisting of…	N	No	?	Yes	N	No	?	Yes	N	No	?	Yes	N	No	?	Yes
(a) offering a listening ear, support and comfort, and being sensible to victims’ immediate practical needs	27	0	0	100	87	1	2	97	26	8	12	81	76	3	9	88
(b) offering practical and up-to-date information about the shocking event	27	0	0	100	87	2	2	95	26	15	15	69	75	5	13	81
(c) mobilising support from the victims’ own social environment	27	0	0	100	87	0	1	99	26	19	12	69	76	1	16	83
(d) facilitating reunion with their nearest and dearest and keeping families together	27	0	4	96	84	1	5	94	26	12	8	81	72	3	22	75
(e) reassuring victims who are displaying normal stress reactions	25	0	4	96	86	0	2	98	25	12	4	84	74	0	12	88

### On preventive early psychosocial interventions

Table 4 shows the results concerning the recommendations on preventive early psychosocial interventions. Interestingly, more than 77% of the respondents are in favour of preventive psycho-education (which is *not* recommended in the Dutch guidelines), and 40% of the European experts and 60% of the Dutch health care professionals consider this to be common practice. During the expert discussions, it was noted that information needs to be appropriate, adapted to specific disasters, to individual situations, and should be appropriately timed (immediate, 6 weeks after, long term). The experts recognised the availability of good practices in almost every EU member state and encouraged sharing of these practices. European and Dutch experts differ on the agreement of the provision of psychological debriefing, be it for victims, relief workers or children. Although EU experts mainly disagree with this intervention (in accordance with recommendations), they do less than the Dutch experts.

**Table 4:** Agreement and practice: preventive early psychosocial interventions (Total N and relative percentages)

	Agreement								Practice							
		
	European Union				Netherlands				European Union				Netherlands			
				
	N	No	?	Yes	N	No	?	Yes	N	No	?	Yes	N	No	?	Yes
Information is offered to those affected consisting of:																
(a) nothing (no information is given)^a^	27	96	4	0	86	83	1	16	26	81	12	8	73	71	12	16
(b) a reassuring explanation about normal reactions	27	4	4	93	88	1	0	99	26	4	12	85	75	4	8	88
(c) explaining when to seek help	27	4	4	93	86	5	2	93	26	8	8	85	73	6	16	78
(d) advising to resume their everyday routine	27	4	4	93	84	4	2	94	26	27	19	54	70	9	19	73
(e) preventive psycho-education^a^	26	15	8	77	78	6	9	85	25	24	32	44	68	15	24	62
Relief workers engage in psychological triage (identification of victims with psychological disorders and/or serious clinical symptoms that need diagnosis and/or treatment)	24	8	4	88	83	2	4	94	23	35	9	57	73	10	15	75
In psychological triage, a distinction is made between (a) victims without mental disorders and/or serious clinical symptoms, (b) victims with regard to whom there is some doubt as to whether they have a psychological disorder and/or serious clinical symptoms (c) victims with mental disorders and/or serious clinical symptoms	27	7	7	85	78	1	9	90	26	27	19	54	63	10	33	57
Peer support is provided	27	4	4	93	85	5	4	92	26	39	12	50	73	8	10	82
To prevent PTSD and other serious disorders in victims, psychological “debriefing” takes place^a^	27	56	15	30	83	84	8	7	26	58	19	23	69	75	17	7
To prevent PTSD and other serious disorders in relief workers, psychological “debriefing” takes place^a^	27	37	4	59	82	63	17	20	26	62	4	35	70	59	29	13
Psychological debriefing is also given to children^a^	25	48	20	32	77	78	16	7	24	67	21	13	69	62	28	10

a Negatively formulated recommendations: these items are inconsistent with the Dutch guidelines.

### On curative early psychosocial interventions

Table 5 summarizes the results on curative early psychosocial interventions as found in the questionnaires. During the expert discussions it was found that these recommendations might require some adaptation for a European audience. First, the timeliness and availability of (certain kinds of) psychosocial support is an area of attention within the EU. In some countries, cognitive behavioural therapy (CBT) is either unavailable or it is not the intervention of choice. Preferred treatment should be adapted to existing national disorder specific guidelines and protocols. Second, the Dutch guidelines currently do not address the issue on how to respond to more complex presentations. Here, a linkage to national guidelines may also be necessary. Third, in expert discussions it was pointed out that it may be necessary to develop a guideline in relation to duty of care for employers following disasters, e.g., for psychosocial care for uniformed persons (rescue workers, military, policemen, firemen, ambulance officers). Finally, the guideline recommendations relating to children currently do not have a strong supporting evidence base. Future research and debate is necessary.

**Table 5:** Agreement and practice: curative early psychosocial interventions (Total N and relative percentages)

	Agreement								Practice							
		
	European Union				Netherlands				European Union				Netherlands			
				
	N	No	?	Yes	N	No	?	Yes	N	No	?	Yes	N	No	?	Yes
Victims with serious symptoms that interfere with their everyday functioning are also offered appropriate symptom- and trauma-oriented therapy (CBT) at an early stage	27	4	15	81	48	6	13	81	25	60	24	16	37	11	30	60
In the event of sleep disorders as a result of a trauma, pharmacotherapy may be considered	27	19	7	74	40	8	18	75	26	27	15	58	37	8	27	65
The employer should offer support and guidance (to be carried out by a relief worker or trained volunteer) when a shocking event takes place at work	27	0	0	100	85	7	1	92	26	62	8	31	69	17	17	65
Ethnic minorities must be approached as normally as possible, but at the same time in a way that is as culture-specific as is necessary. The latter consists of providing information in their mother tongue and involving key figures from ethnic minority groups	25	0	4	96	80	6	5	89	24	58	4	38	65	20	34	46
Relaxation is offered as a separate intervention (not trauma-oriented)^a^	27	30	22	48	38	29	32	40	26	73	15	12	35	24	38	38

a Negatively formulated recommendations: these items are inconsistent with the Dutch guidelines.

### On the organisation of early psychosocial care

Table 6 shows the questionnaire results on the organisation of early psychosocial care. The broad consensus on the relevance of these topics was confirmed in the expert discussions. The attention from governments, politics and policy makers for psychosocial interventions will vary within different member states. This underscores the importance of developing an integrated approach across health structures, policies and political strategies on a national level. It is equally important to create strong and clear leadership in both social and clinical disaster responses, to educate local partners and to engage with communities to help them respond supportively.

**Table 6:** Agreement and practice: the organisation of early psychosocial care (total N and relative percentages)

	Agreement								Practice							
		
	European Union				Netherlands				European Union				Netherlands			
				
	N	No	?	Yes	N	No	?	Yes	N	No	?	Yes	N	No	?	Yes
Early psychosocial interventions must be provided by (specially) trained persons	27	4	4	93	80	8	0	93	26	27	12	62	68	10	12	78
Community-level interventions form an essential part of the whole package of post-disaster psychosocial care	27	0	0	100	78	5	9	86	26	39	23	39	62	8	29	63
In the first 6 weeks after a disaster a good system of relief is established	26	12	12	77	79	3	5	92	25	36	24	40	63	5	30	65

## Discussion

This study explored whether experts within Europe agree on a set of recommendations on early psychosocial intervention after shocking events (the Dutch guidelines), and to what degree these standards are considered to actually be brought into practice. Among both European experts and Dutch health care professionals, a (very) high level of agreement on the recommendations was found. However, the degree to which these recommendations were actually brought into practice clearly lagged behind. These two main outcomes are discussed in more detail below.

### Overall consensus on early psychosocial interventions; some discrepancies

European and Dutch experts agree on the aims of the Dutch guidelines, some of the screening interventions, the provision and components of a supportive context, the provision of information to those affected by disaster, trauma focused CBT as a treatment for acute stress disorder and PTSD, the provision of professional counselling by employers in case of work related shocking events, dealing with ethnic minorities, and the organisation of early psychosocial care. In a more general sense, the experts agree that the Dutch guidelines can serve as a source of “guidance” in the development of national guidelines within other countries.

Issues where there was less agreement included the use of PTSD-questionnaires for screening, the necessity of community-level interventions and the timely establishment of a system of relief. Another notable difference was found relating to the agreement on the provision of psychological debriefing, be it for victims, relief workers or children. Although EU experts in the majority disagree with this intervention, they do less so than Dutch experts. Psychological debriefing can be described as a standardized crisis intervention, the purpose of which is to prevent and reduce the adverse psychological effects of traumatic events. Although it takes many different forms, it is generally seen as a once-only, semi-structured intervention. Research has indicated that psychological debriefing after a shocking event is not effective in preventing PTSD and other psychological problems and that single-session debriefing can even have damaging effects (e.g., Aulagnier, Verger, & Rouillon, 2004; Lewis, 2003; Van Emmerik, Kamphuis, Hulsbosch, & Emmelkamp, 2002; Sijbrandij et al., 2007). Literature does not support the use of psychological debriefing for children either (Stallard et al., 2006). As a result, the Dutch guidelines do not recommend the use of once-only psychological debriefing. Differences in interpretations of what psychological debriefing actually constitutes might in part explain the differences between EU and Dutch experts. Other explanations may be of a more practical nature, e.g., there may be conflicts of interests resulting from practitioners having a vested interest in professional counselling provided by employers. Such differences, especially when they are deeply rooted in everyday practice, may seriously hamper the implementation of new insights, and thereby, widen the gap between norms and practice.

Interestingly, on a number of recommendations European and Dutch experts “agree to disagree”. There is, for instance, agreement that an increased risk to develop PTSD can be identified by either using PTSD questionnaires as well as the diagnosis of Acute Stress Disorder – both of which are *not* recommended by the Dutch guidelines. In the same vein, psycho-education, consisting of structured information and training to those affected, is not recommended by the Dutch guidelines, as there is no scientific support for its effectiveness (e.g., Ehlers & Clark, 2003a; Ehlers et al., 2003b; NICE, 2005; Turpin, Downs, & Mason, 2005; Sijbrandij et al., 2007). Nevertheless, both groups of experts indicate that preventive psycho-education should be used. This finding also could be due to differences in the definition of psycho-education and a possible overlap with the provision of information in itself (which is recommended and agreed upon). In summary, the level of agreement on the recommendations in the Dutch guidelines is substantial, although there remain issues that require additional specification for their “translation” to countries outside The Netherlands.

### Closing the gap between norms and practice

The necessity for effective guideline implementation is further underscored by the second main outcome of his study: the expected gap between norms and actual practice appears to exist. Interestingly, even when in agreement with the recommendation not to use certain practices, experts indicate it is part of daily practice. The discrepancy between “ought” and “is” was notable among European experts and Dutch professionals. If this gap is to be closed, it needs to be fully understood. It is essentially caused by three conditions: motivation, capability and opportunity (see, e.g., Michie, van Stralen, & West , 2011). Motivation has to do with knowing what is recommended and being enthusiastic about acting as such. Capability is reflected in the presence of the right knowledge, skills, competencies, talents, etc. to act. Having the opportunity to do so depends on external factors, e.g., time, resources and organizational support. This latter point was also raised by the European experts in addressing the variance in developmental stage of psychosocial care in different member states – not all countries are able to provide adequate support and resources to create appropriate opportunity to actually implement norms into practice.

In his discussion of organizational readiness for change, Weiner (2009) states that although the professional's organization must be ready for change, the professional's motivation is crucial for successful implementation. What is relevant here is that professionals providing psychosocial care are characterized by a relatively large degree of discretionary space and autonomy, which is typical for the classical street-level bureaucrat (Lipsky, 1980). In their dialogue with individual victims or clients, professionals may well arrive at treatment that differs from the care delivery strictly advocated by guidelines. Those confronted with a shocking event might demand something which is, strictly considered, not regarded as effective, but nonetheless considered acceptable, not to say beneficial, by the professional involved. This professional autonomy is a further explanation for the chasm. Although the Dutch guidelines leave room for the professional's ad hoc choices, any deviation from guidelines should always be substantiated with arguments.

This study revealed a universal gap between psychosocial care principles and practice. Bridging it is a major challenge. Defining standards and norms is a starting point, but every general standard needs to be translated to the local context where it will be used. To overcome cultural discrepancies and idiosyncrasies, it is crucial to involve stakeholders and interest groups in knowledge translation. Also, specific target groups and contexts can be identified that require specific guidelines (e.g., uniformed emergency services, ethnic groups or children). Making a distinction in target groups is also beneficial for implementation, especially because the principles that are to be implemented, affect people at the coalface, dealing with real problems and issues in providing psychosocial care. Closing the gap encompasses extensive tailoring and consideration of context-specific needs and interests (Greenhalgh, Robert, Macfarlane, Bate, & Kyriakidou, 2004; Vos, Dückers, Wagner, & Van Merode, 2010). Ideally, this would be supported by a systematic meta-review of existing international guidelines to compare assumptions, interventions, evidence strength, and the extent to which success factors for the implementation are taken into account (as described by Eccles et al., 2012).

### Limitations

Although attempts were made to find an accurate representation of mental health care givers in the selection of experts from the EU, an obvious limitation of this study is that the samples used are not randomly drawn. Adequate sampling of persons involved in the provision of psychosocial care after disasters is hampered by the fact that this topic does not receive the same amount of attention in all European countries. The Dutch sample could also be conceived of a convenience sample, given its non-random composition. This increases the possibility of a sampling bias and makes it less tenable to generalize results to “an ideal population”. However, given the above-mentioned differences in the different countries in the way early psychosocial health care is organized, one would be hard-pressed to adequately describe such an ideal population (let alone obtaining a random sample from it). Furthermore, sample characteristics comprise (mental) health care professionals in general, including policy makers, (acute) caregivers, and volunteers – the Dutch guidelines are relevant for all these groups.

Nevertheless, the main findings of this study (i.e., a large overall agreement to the recommendations of the Dutch guidelines, and a noticeable gap between theory and practice) should be replicated in follow-up research, preferably within the separate EU states and (where possible) using an adequate sample reflecting the national status quo. This way, the differences found in this study can be more thoroughly investigated, and the effects of processes that can occur in group discussions (e.g., “groupthink”) can be excluded. Where does the apparent gap between theory and practice appear the most, and why? Questions like this are especially relevant concerning the discrepancies found on screening (including the use of PTSD questionnaires), psychological debriefing, and psycho-education.

## Conclusion

As stated in the introduction, there is increased attention on academization of the mental health field (Engelhart, 2012). This study shows that, in the case of early psychosocial interventions, valorisation, i.e., the translation of academic knowledge into practice, does not seem to be hindered by the acknowledgement of the knowledge itself. Therefore, there are other issues that hamper closing the bridge between theory and practice. Although these issues are touched upon in this study, they deserve better understanding. The results of this exploratory study reflect opinions of individual European experts, and more research is necessary to confirm findings. Nevertheless, the expected discrepancy between norms and practice appears to exist throughout the EU, stressing the need for investments in guideline development and implementation.

## Footnotes

1 European guidelines for target group oriented psychosocial aftercare. Project co-funded by the European Commission – Grant Agreement No. 07. Project co 030601/2006/447903/SUB/A3.
